# Single digit micronychia in an infant

**DOI:** 10.1016/j.jdcr.2025.01.008

**Published:** 2025-01-31

**Authors:** Jacob T. Tribble, Rachel Fayne, Benjamin M. Mervak, Julie E. Mervak

**Affiliations:** a University of Missouri-Kansas City School of Medicine, Kansas City, Missouri; b Department of Dermatology, University of Michigan, Ann Arbor, Michigan; c Department of Radiology, University of Michigan, Ann Arbor, Michigan

**Keywords:** congenital, micronychia, newborn, onychodysplasia

## Case presentation

**Fig 1:**
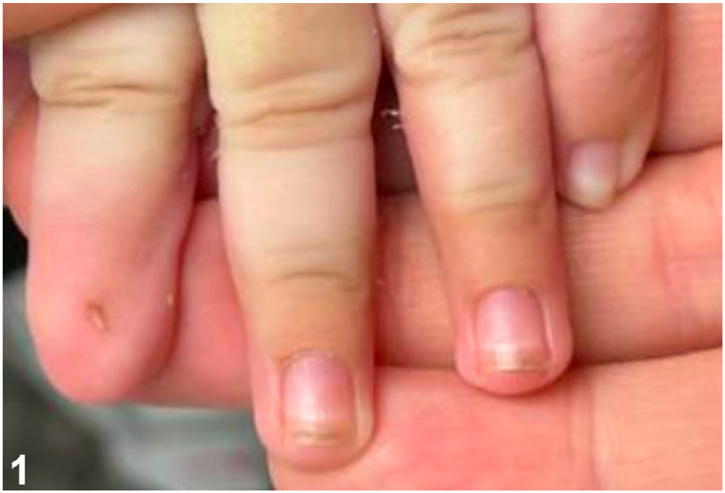

A two-month-old male presented to dermatology clinic for evaluation of nail dystrophy of the left second fingernail noted at birth. He was a full-term newborn and, apart from prenatal vitamins, his mother denied any medication or drug exposure during pregnancy. Physical exam revealed micronychia isolated to the left second fingernail (Fig 1) with normal appearance of other fingernails and toenails. Family history was negative for similar findings, including 2 older sisters.

**Question 1: What is the most likely diagnosis?**

A. Congenital hypertrophy of the lateral nail folds
B. Congenital onychodysplasia of the index finger (COIF)
C. Ectodermal dysplasia
D. Nail-patella syndrome
E. Pachyonychia congenita

**Answers:**

A. Congenital hypertrophy of the lateral nail folds – Incorrect. This is a nail finding in newborns most often affecting the first toenail. The lateral nail fold covers a portion of the nail plate, giving the appearance of micronychia.^1^ This resolves in the first year of life.
B. Congenital onychodysplasia of the index finger (COIF) – Correct. Iso-Kikuchi syndrome, subclassified as COIF when involving the index (second) fingernail, presents at birth with unilateral or bilateral anonychia, micronychia, polynychia (rudimentary splitting), hemionychogryphosis, nail malalignment, or an irregular lunula of the nail.^2^ The etiology is unknown; however, several theories have been proposed, including mutations in the Wnt signaling pathway, in utero damage to arterioles of the radial artery, or osteodystrophy of the phalanx during fetal development.^2^
C. Ectodermal dysplasia – Incorrect. Nail hypoplasia can be observed in ectodermal dysplasia disorders. However, ectodermal dysplasias also present with hair, teeth, and sweat gland abnormalities. Nail changes involve multiple digits and are not specific but can include micronychia with thickening or brittle nails.^1^
D. Nail-patella syndrome – Incorrect. Nail-patella syndrome presents with anonychia or micronychia, along with triangular lunulae, which is commonly bilateral and symmetrical, with severity most pronounced on the thumbs (first fingernails) and less severe moving toward the fifth fingernails.^1^
E. Pachyonychia congenita – Incorrect. Pachyonychia congenita presents with nail thickening and increased curvature, not anonychia or micronychia. Nail changes become evident around 2 to 3 years old. Often, the first and second fingernails are most severely affected; however, in some patients, toenails are more significantly involved.^1^

**Question 2: Although often a clinical diagnosis, which next step could be considered?**

A. Magnetic resonance imaging of the left hand
B. Radiographs of the left hand
C. Radiographs of the bilateral lower extremities and pelvis
D. Referral to genetics
E. Referral to pediatric dentistry

**Answers:**

A. Magnetic resonance imaging of the left hand – Incorrect. COIF does not present with any abnormalities to the soft tissues (muscles, ligaments, tendons) of the hand. Therefore, detailed imaging with magnetic resonance imaging is not indicated.
B. Radiographs of the left hand – Correct. While not always present, radiographic findings in COIF can include Y-shaped deformity of the affected distal phalanges (best seen on lateral radiographs) or narrowing of the distal phalanx (best seen on anteroposterior view). The presence of characteristic radiologic findings has been reported as early as in infancy.^3^ In this patient, no abnormalities were present.
C. Radiographs of the bilateral lower extremities and pelvis – Incorrect. Bone findings in nail-patella syndrome include luxation or complete absence of the patella, arthrodysplasia of the elbow, and the presence of bilateral accessory iliac horns.^1^ No pelvic abnormalities are seen in COIF and given normal toenails; no bony abnormalities of the feet were expected in this patient.
D. Referral to genetics – Incorrect. Although in the diagnostic criteria for COIF there is mention of possible autosomal dominant transmission,^2^ most cases of COIF appear to be sporadic, and no specific genetic mutation has been identified. Given the isolated abnormality of the nail and no association with systemic abnormalities, genetic testing can be deferred.
E. Referral to pediatric dentistry – Incorrect. Unlike ectodermal dysplasias, there is no association with teeth abnormalities in COIF.

**Question 3: Which of the following maternal medications, if elicited in the prenatal history, would be most likely to cause micronychia in a newborn?**

A. Methimazole
B. Phenytoin
C. Tetracycline
D. Topiramate
E. Warfarin

**Answers:**

A. Methimazole – Incorrect. Fetal exposure to methimazole is associated with aplasia cutis,^4^ but not nail abnormalities.
B. Phenytoin – Correct. Exposure to antiepileptic medications such as phenytoin, phenobarbital, and carbamazepine has been linked to congenital nail deformities. Phenytoin is particularly implicated as a teratogenic medication. Along with nail and digital hypoplasia, newborns who were exposed to phenytoin can have microcephaly, congenital heart defects, cardiac rhythm disturbances, renal dysplasia, and gastrointestinal abnormalities, all of which are grouped under the findings of fetal hydantoin syndrome.^5^
C. Tetracycline – Incorrect. Tetracycline class antibiotic exposure in utero is linked to discoloration of bones and teeth with high dose suppressing skeletal growth and tooth enamel hypoplasia.^4^
D. Topiramate – Incorrect. Topiramate has been linked to hypospadias and oral clefts in newborns, not with anonychia or micronychia.^4^
E. Warfarin – Incorrect. Warfarin use during pregnancy has been associated with fetal skeletal abnormalities, stippled calcification of epiphysis, and nasal hypoplasia but not specifically nail dysplasia.^4^

## Conflicts of interest

None disclosed.
